# Media Exposure and General Trust as Predictors of Post-traumatic Stress Disorder: Ten Years after the 5.12 Wenchuan Earthquake in China

**DOI:** 10.3390/ijerph15112386

**Published:** 2018-10-27

**Authors:** Lingnan He, Kaisheng Lai, Zhongxuan Lin, Zhihao Ma

**Affiliations:** 1 School of Communication and Design, Sun Yat-sen University, Guangzhou 510006, China; heln3@mail.sysu.edu.cn (L.H.); lzhongx55@sina.com (Z.L.); 2 Guangdong Key Laboratory for Big Data Analysis and Simulation of Public Opinion, Guangzhou 510006, China; 3 School of Journalism and Communication, Jinan University, Guangzhou 510632, China; kaishenglai@126.com; 4 Computational Communication Collaboratory, School of Journalism and Communication, Nanjing University, Nanjing 210023, China

**Keywords:** media exposure, general trust, post-traumatic stress disorder, earthquake, China, Sichuan/Wenchuan

## Abstract

There is a paucity of literature on the roles of media exposure, general trust, and their interactions in long-term post-traumatic stress disorder (PTSD) symptoms after a natural disaster. Trying to address this knowledge gap, our study aimed to (a) investigate whether exposure to media coverage during the traumatic event and general trust directly affected adult survivors’ long-term PTSD symptoms 10 years after the 5.12 Wenchuan earthquake, and (b) to identify the potential differential pattern of the influence of media exposure on PTSD symptoms for adult survivors with various levels of general trust. Using cross-sectional methodology, we surveyed participants (N = 1000) recruited from six disaster-affected counties. We assessed PTSD symptoms, media exposure, general trust, demographic characteristics, socioeconomic status, and earthquake exposure. Data were analyzed descriptively and with Tobit regression analyses. Reversed relationships between general trust and PTSD were verified, whereas no direct links were found between media exposure and PTSD. Interaction tests revealed that media exposure alleviated PTSD for high-trust survivors, but aggravated PTSD for low-trust survivors. These results suggest that general trust building should be considered in post-disaster construction activities.

## 1. Introduction

From 2006 to 2015, China had the highest number of people (1,019,008,563) affected by disasters in the world [1]. This comprises 53% of disaster victims globally over the same period [1]. The 5.12 Wenchuan earthquake was the worst disaster that occurred in these years. The disaster caused 845.1 billion China Yuan (over 100 billion US dollars) in direct economic losses [2], 69,227 people died, 374,643 people were injured, and 17,923 people were missing [3]. As one typical and common mental disorder arising after exposure to natural disasters [4], post-traumatic stress disorder (PTSD) has been a key concern for both scholars and policymakers in the recent three decades [5,6,7,8]. One meta-analysis revealed that the prevalence of PTSD after earthquakes ranged from 4.10–67.07% in adults and from 2.50–60.00% in children [9]. It also demonstrated that being a woman, having lower socioeconomic status, and some earthquake exposure indicators were significant risk factors for developing PTSD symptoms [9]. Recently, as predictors of PTSD, media exposure and general trust have also received scholars’ attention when examining the mental health consequences of terrorist incidents and regional wars [10,11,12]. Consequently, there is a need for an insightful framework to verify whether media exposure and general trust play key roles in the mental health consequences associated with the 5.12 Wenchuan earthquake.

PTSD was the most prevalent mental disorder for both rescuers and survivors of the 5.12 Wenchuan earthquake [13,14,15]. The prevalence of PTSD among adult survivors was reported as 62.8% in Qingchuan one month after the earthquake [16], 43% in Mianzhu two months after the earthquake [13], and 37.8% and 13.0% in two temporary camp communities, respectively, in Beichuan three months after the earthquake [15]. In addition, children and adolescent survivors were affected by PTSD [17]; however, the results varied per survey site and among participants. One longitudinal study indicated that the prevalence of PTSD and PTSD symptoms declined over time [18]; however, PTSD may have lifelong persistence [19]—another study revealed that 29.6% of adolescent participants reported clinical symptoms of PTSD three years after the 5.12 Wenchuan earthquake [20]. Even for some adult survivors, PTSD was still highly prevalent for years. One study conducted in Beichuan eight years after the earthquake revealed that the prevalence of symptomalogical PTSD was 11.8% [16].

Ten years have passed since the earthquake, and many survivors may still have mental disorders. More research on the risk factors of long-term psychiatric outcomes is needed. Demographic variables, socioeconomic status, and disaster exposure are the most commonly considered risk factors to predict PTSD in traditional epidemiological and psychiatric approaches [9]. These factors are robust for short-term PTSD symptoms; however, they are inadequate for predicting long-term psychopathological consequences since people are assumed to be living with protentional lifelong exposure to traumatic events in today’s informational society [21].

Linking exposure to media coverage of traumatic events and psychopathological consequences is one rational approach to understanding antecedents of long-term mental health effects of disasters [21]. There are two potential mechanisms to explain the role of media coverage in forming PTSD. The first is secondary traumatization, which implies that people may reexperience the traumatic event via media coverage, thus leading to mental disorders regardless of direct exposure to the event [22]. Following the events of September 11th in the United States, several studies indicated that media is the most common trigger of memory recall to evoke people’s psychopathological outcomes [23,24,25,26]. Ahern and colleagues found that exposure to television coverage during the attack was strongly associated with viewers’ PTSD symptoms four months after 11 September [23]. Silver and colleagues reported that media exposure behavior even predicted increased PTSD symptoms two to three years after 11 September [24]. Contrarily, the second mechanism is stress alleviation, which refers to people trying to seek more information about the traumatic event to reduce the perception of uncertainty and to alleviate stress [27,28]. Exposure to media coverage about the traumatic event is one typical coping strategy when people have harmful mental consequences after a traumatic event. A previous study implied that seeking information about September 11th may reduce negative emotion after the attack [27], whereas little evidence was found to support the assumption that media exposure relieved psychological stress. This finding raised the question of whether media exposure during the traumatic event is indeed traumatic, or if it acts as a mental buffer, and whether the PTSD symptoms associated with media exposure persist 10 years after the 5.12 Wenchuan earthquake for adult survivors. Given the link between media exposure and mental disorder outcomes that consistently emerges in the study area of PTSD after terrorist events, we hypothesized the following:

**H1:**  Exposure to media coverage about the traumatic event during the disaster will be associated with PTSD symptoms among adult survivors 10 years after the 5.12 Wenchuan earthquake.

Another approach to predicate long-term PTSD symptoms is based on the social capital framework. As one umbrella concept of social connection process, social capital is a topic increasingly gaining attention from epidemiologists and political scientists to understand and improve mental health outcomes [29,30,31], including PTSD [5,32,33]. Social capital is also one crucial theoretical framework to operate disaster-related risk management [34,35]. Studies demonstrate that social capital was associated with better performance of decision-making process in public disaster protection projects and more efficient risk campaigns [35,36].

Particular attention is paid to one important component—general trust [37]—which refers to trust in non-specific others [38], and its role in ameliorating disaster victims’ mental disorders [5], increasing perceived self-efficacy [35], and providing social support during and after a disaster [35]. General trust was also shown to be positively associated with reduced health risk behaviors (e.g., smoking, alcohol drinking) [30,39], increased self-rated health [40], and palliated PTSD symptoms among survivors four years after an earthquake [5]. More importantly, one recent study revealed that general trust had a strong relationship with lifelong PTSD symptoms [32]. Scholars emphasize that strategies and intervention options for repairing trust for affected people are needed [32]. In the case of the 5.12 Wenchuan earthquake, it is possible that general trust could help alleviate long-term PTSD symptoms among disaster victims. Consequently, we hypothesized the following:

**H2:**  General trust will be negatively associated with PTSD symptoms among adult survivors 10 years after the 5.12 Wenchuan earthquake.

This study has focused thus far on two factors associated with both individual and collective post-disaster coping strategies—media exposure and general trust—and their potential direct effects on long-term PTSD symptoms. However, in addition to drawing direct connections between these predictors of PTSD symptoms, previous studies also suggest that issues related to social trust were primary factors to explain how exposure to media coverage about risk events amplifies the social risk [41,42]. If disaster victims do not trust the media coverage during a disaster, their risk perception, reactions toward the disaster, and uncontrollability of subjective consciousness will be strengthened [41]. Hence, the role of trust may differ in various media environments. Moreover, the effects of information exchange on the risk perception of a disaster, which may be associated with increased mental disorders [43], were also moderated by victims’ attitude towards specific risks [44]. These prior findings raised the question of whether these two concerned factors will be interactively associated with mental health outcomes.

The existing literature has yet to establish a theoretical framework to determine whether media exposure amplifies or diminishes the role of general trust in relieving PTSD symptoms among disaster victims. Further, empirical research has not investigated the buffering effect of general trust on exposure to media coverage of traumatic events in a mental health context. General trust is generally regarded as a social determinant of mental health, whereas the role of media exposure has resulted in inconsistent findings. Consequently, and given the complicated situation concerning the post-disaster rebuilding after the 5.12 Wenchuan earthquake, this study exploratorily assumes that general trust moderates the effects of media exposure on PTSD symptoms. We tested this with the following question:

**RQ:**  Does how media exposure affects PTSD symptoms differ between adults who have a high level of general trust and those who have a low level of general trust?

## 2. Materials and Methods

### 2.1. Sampling Procedures

**Figure 1:**
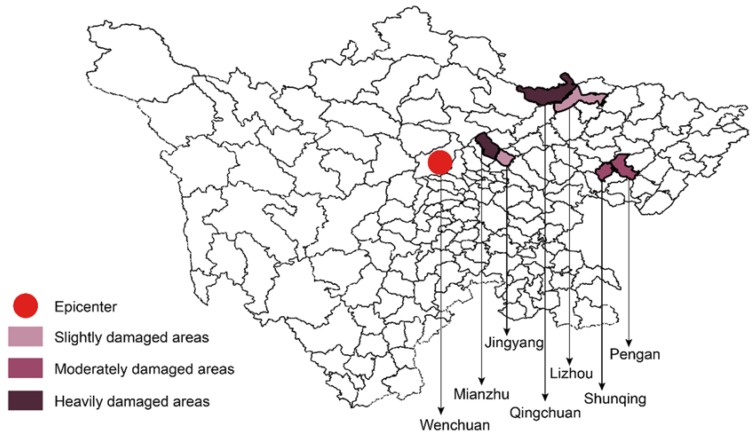
Map of survey regions.

The survey team conducted a cross-sectional study across six counties in Sichuan in May 2018—the 10th anniversary of the 5.12 Wenchuan earthquake. The Chinese government divided all damaged areas into three categories: Heavily damaged, moderately damaged, and slightly damaged [45]. The six counties this study (see Figure 1) selected were two slightly damaged areas (Jingyang and Lizhou), two moderately damaged areas (Shunqing and Pengan), and two heavily damaged areas (Mianzhu and Qingchuan). In the initial field investigation, the survey team selected 26 communities according to the damage situations and residents’ living arrangement after the earthquake. In the second stage of sampling, 30 to 50 individuals were selected within each settlement according to the local population size. Overall, data from 1000 adult survivors were included. Inclusion criteria were as follows: All participants were already adults and lived in the local county before the earthquake, and they did not migrate to other places longer than one year after the earthquake.

### 2.2. Measures

#### 2.2.1. PTSD Symptoms

The PTSD Check List–Civilian Version (PCL-C) was used to assess PTSD symptoms [46]. This instrument consists of 17 items that were developed according to the diagnostic criteria of PTSD set by the Diagnostic and Statistical Manual of Mental Diseases, fourth edition [46,47]. It presents three symptoms: Reexperiencing, avoidance, and arousal. The PCL-C has been shown to be reliable in diverse languages and has been validated in many countries, including China [48]. In this study, each item was measured from 1 to 5 (1 = not at all bothered, 2 = slightly, 3 = moderately, 4 = severely, and 5 = extremely severely). In this study, Cronbach’s alphas were 0.898 for the PCL-C, 0.712 for reexperiencing, 0.774 for avoidance, and 0.780 for arousal. Since we examined the links between media usage, general trust, and PTSD symptoms, the PCL-C overall score and its three sub-scores were calculated by the mean of related items.

#### 2.2.2. Media Exposure

Based on the suggestions of previous researchers [49,50], media exposure was measured by a one-item media exposure scale. All participants were asked to estimate how many times they spent watching the news about the earthquake via media platforms (e.g., newspaper, broadcasting, television, Internet, etc.) in the following month after the 5.12 Wenchuan earthquake. Responses ranged from 1 (never) to 5 (a lot).

#### 2.2.3. General Trust

General trust was also evaluated via a widely used one-item general trust scale [30,51]. All participants were asked to appraise the item, “Do you approve that most people in this society can be trusted?” Responses comprised the following: “do not agree”, “neutral”, “agree”, and “do not know.” This variable was dichotomized into “high trust” (1) and “low trust” (0), with the response, “agree”, being the high trust group. Other responses were combined and given a 0 value.

#### 2.2.4. Control Variables

We treated demographic variables, socioeconomic status, and earthquake exposure as control variables. Data collected included participants’ gender, age, community status, marital status, educational background, annual household income, and 16 items about direct/indirect earthquake exposure developed by previous studies [52,53].

### 2.3. Statistical Analysis

**Figure 2:**
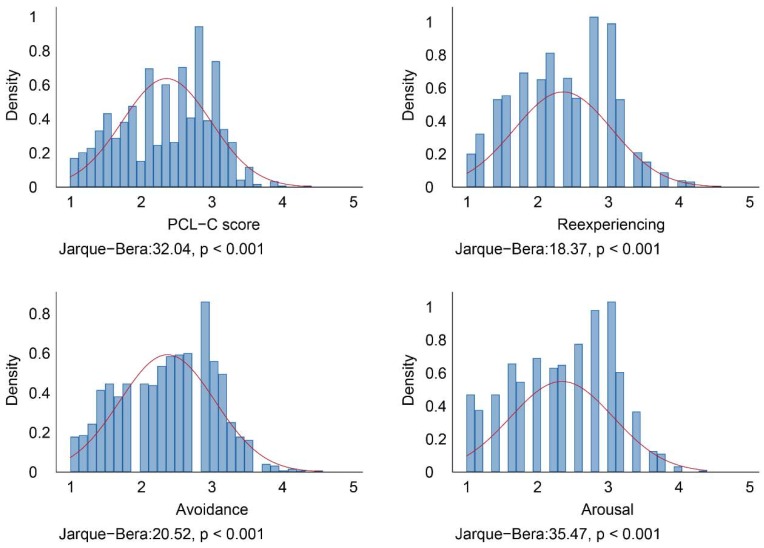
The Jarque–Bera statistic tests for the null hypothesis of normality for the distribution of the series.

Most previous studies estimated via the ordinary least squares strategy; however, there are definite boundaries (from 1 to 5) for dependent variables. In addition, the Jarque-Bera statistic (see Figure 2) demonstrates that the null hypothesis of normality is rejected at the 1% significance level [54]. These imply that regression models may be biased estimated via the ordinary least squares strategy.

Hence, this study adopted the Tobit regression model [55] to make this estimation as follows:

(1) $$\begin{array}{r} PTSD_{i}^{*}=\beta_{0}+\beta_{1}Media_{i}+\beta_{2}Trust_{i}+\beta_{3}X_{i}+\varepsilon_{i}\\ PTSD_{i}=\{\begin{array}{cc} 5 & if\;PTSD_{i}^{*}>5\\ PTSD_{i}^{*} & if\;1<PTSD_{i}^{*}\leq 5\\ 1 & if\;PTSD_{i}^{*}\leq 1 \end{array} \end{array}$$

$PTSD_{i}$ in Equation (1) shows the estimated PTSD status for individual *i*; $PTSD_{i}^{*}$ is the direct expected value for observations; $Media_{i}$ is the media exposure status for individual *i*; $Trust_{i}$ is the general trust for individual *i*; $X_{i}$ is the vector of control variables; *ε_i_* is the independently distributed error term; and *β_1_* and *β_2_* are target coefficients, which will be estimated for testing two research hypotheses. 

To answer the research question, “How media exposure affects PTSD symptoms differ between adults who have a high level of general trust and those who have a low level of general trust?”, we added an interaction item into the estimation:

(2) $$PTSD_{i}^{*}=\alpha_{0}+\alpha_{1}Media_{i}+\alpha_{2}Trust_{i}+\alpha_{3}Media_{i}*Trust_{i}+\alpha_{4}X_{i}+\omega_{i}$$

$Media_{i}*Trust_{i}$ in Equation (2) is the interaction item of media exposure and general trust for individual *i*, and *α_3_* is the target coefficient that will provide the evidence about this research question.

## 3. Results

### 3.1. Participants’ Characteristics

Table 1 shows descriptive statistics results for a variety of measures of PTSD symptoms, independent variables, demographic and socioeconomic variables, and earthquake exposure. Overall, 43.5% were male, 55.7% were living in rural sites, 82.7% were married, and the mean age was 46 years (SD = 12 years). The minority of participants had attended associate college and above (25.3%). Further, the proportion of higher annual household income (90,000 RMB and above) was low (22.1%). The PCL-C score and three PTSD symptoms scores were less than 3, which is the average score of the cut-off point to identify PTSD. Participants showed a moderate amount of media exposure in the following month after the earthquake. Additionally, 57.5% displayed a high level of general trust. For the earthquake exposure situation, most participants experienced moderate (42%) to serious (31%) loss of property. The prevalence of the other 15 indicators of earthquake exposure ranged from 1.8% (being disabled) to 42.3% (acquaintance injured).

### 3.2. Main Effects

Table 2 provides an overall picture of the main effect of the regression estimates. Prior to reporting the results of our hypotheses tests, we first illustrate the Tobit estimates of PCL-C score and three PTSD symptoms, based on demographic variables, socioeconomic status, and earthquake exposure status for all 1000 valid participants (via Models 1, 3, 5, and 7 of Table 2).

There was also a strong education gradient: Higher education corresponded to increased PCL-C scores and three PTSD symptoms. This finding contradicts one recent meta-analysis [9]. One likely explanation is that the long-term PTSD symptoms may be diminished over time much easier for people with less education than their higher educated counterparts. For those higher educated survivors, the buffer effect of education on mental disorders may be extruded.

Earthquake exposure related indicators show complicated relationships with PTSD symptoms. Being injured was negatively related to PCL-C score (*β* = −0.13, *p* < 0.05), avoidance (*β* = −0.14, *p* < 0.05), and arousal (*β* = −0.17, *p* < 0.05); family injured was positively related to PCL-C score (*β* = 0.12, *p* < 0.05), reexperiencing (*β* = 0.19, *p* < 0.01), and arousal (*β* = 0.12, *p* < 0.1); family disabled was negatively related to reexperiencing (*β* = −0.17, *p* < 0.1); kinsfolk died was negatively related to all outcome variables (*β*s ranged from −0.19 to −0.11, all *p*s < 0.1); kinsfolk disabled was negatively related to reexperiencing (*β* = −0.11, *p* < 0.1); acquaintance died was positively related to PCL-C score (*β* = −0.12, *p* < 0.05), avoidance (*β* = −0.14, *p* < 0.01), and arousal (*β* = −0.14, *p* < 0.05); witness to others’ burial was negatively related to PCL-C score (*β* = −0.11, *p* < 0.1) and reexperiencing (*β* = −0.13, *p* < 0.05); and witness to others’ death was negatively related to PCL score (*β* = −0.09, *p* < 0.1), avoidance (*β* = −0.10, *p* < 0.05), and arousal (*β* = −0.12, *p* < 0.05). Participants who reported higher levels of house and property loss had worse PTSD symptoms than those with lower levels.

There were not any coefficients of media exposure in Models 2, 4, 6, or 8, as shown in Table 2, which shows significant results (*β*s ranged from −0.03 to 0.01, all *p*s > 0.05). Hence, H1 was not supported. Further, general trust was significantly negatively associated with PCL-C score (*β* = −0.12, *p* < 0.01), reexperiencing (*β* = −0.08, *p* < 0.1), avoidance (*β* = −0.13, *p* < 0.01), and arousal (*β* = −0.16, *p* < 0.001), indicating general trust is a key predictor of long-term PTSD symptoms among adult survivors 10 years after the 5.12 Wenchuan earthquake. Thus, H2 was statistically corroborated.

### 3.3. Interaction Effects

Table 3 shows the results of the Tobit estimates of the PCL-C score and three PTSD symptoms, with an extra predictor of the interaction item of media exposure and general trust to answer the research question proposed above. Coefficients of the interaction item in Model 1 (*β* = −0.07, *p* < 0.1) and Model 4 (*β* = −0.10, *p* < 0.05) were negatively, marginally significant, which demonstrates that, compared with participants who had low trust, PCL-C score and arousal among participants with high trust were mitigated via exposure to media coverage about the disaster during the earthquake. Whereas, the interaction item in Models 2 and 3 was not significantly related to the outcome variables. 

**Figure 3:**
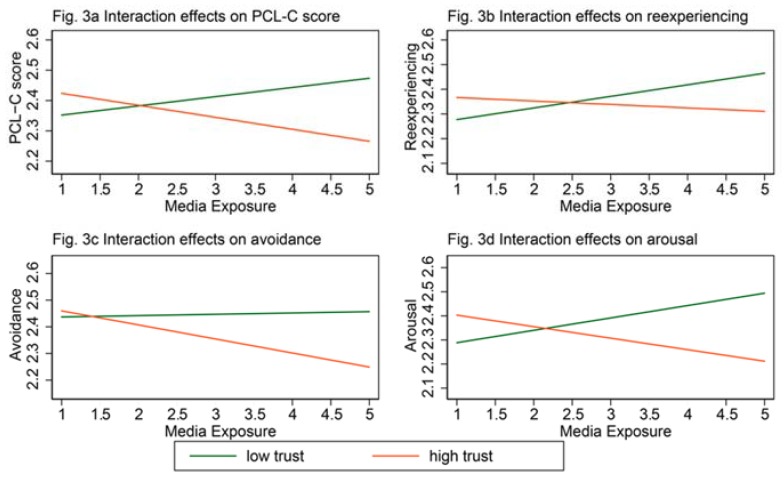
Interaction effects of media exposure and general trust on PTSD symptoms.

To observe detailed information of these moderating effects, Figure 3 shows patterns of interaction effects with slope variations for people with low trust and people with high trust separately (separate Tobit regression results can be found in Table A1 of Appendix A). Figure 3a shows a negative slope for high trust participants and a positive slop for low trust participants. This pattern reveals that media exposure during the disaster alleviated the PCL-C score for high trust survivors, whereas it aggravated low trust survivors’ PCL-C score. Figure 3b shows a similar pattern—the most significant difference is that the line of high trust participants is much flatter, which means that media exposure slightly decreases the reexperiencing symptom for high trust survivors. Figure 3c shows a reversed pattern—the line of low trust participants is flat, which indicates that media exposure does not correlate with the avoidance symptoms of low trust survivors. Figure 3d also shows a similar pattern with Figure 3a—for high trust survivors, arousal symptoms decreased with more exposure to media coverage about the disaster during the earthquake; whereas, for low trust survivors, increased media exposure corresponded to a significant increase in arousal symptoms.

## 4. Discussion

To the best of our knowledge, this is the first study concerning media exposure and general trust as predictors of PTSD symptoms in a natural disaster context. It also provides sophisticated information to understand the psychopathological consequences of the 5.12 Wenchuan earthquake for adult survivors 10 years later.

Regarding media exposure, we found no direct link between media exposure to coverage of the disaster and long-term PTSD symptoms. This is inconsistent with most previous studies, which demonstrated positive relationships between media exposure and worse psychopathological consequences [56,57,58]. There are two possible explanations for this inconsistency. One simple explanation would be that the media exposure during the disaster may affect survivors’ mental outcomes in a short-term period, whereas, these direct linkages cannot persist for 10 years. The second explanation is that, during the disaster, survivors lived in temporary shelters and experienced fear, anxiety, and depression [20,59]. Media exposure is just one coping strategy to reduce information uncertainty, which can be motivated by a short-term mental disorder and directly affected by earthquake exposure [15,17]. Certainly, there was some conclusive evidence revealed in the interaction tests, which will be discussed shortly.

As predicted, associations between general trust and long-term PTSD symptoms were strongly reversed. These results are consistent with previous studies that demonstrated a positive relationship between general trust and individual health outcomes [32,60]. General trust is a typical social interaction indicator of modern civil society [61], whereas traditional Chinese society has a differential pattern of trust that implies that the trust usually has hierarchical variations according to whether people have a kinship relationship [62]. Scholars believed the differential pattern of trust derived from traditional Confucians culture, which was verified as leading to worse general trust [63]. In the post-disaster construction process, traditional social norms and social connections were modified by outside forces and resources. New roles for the general trust have emerged taking precedence over traditional norms. People with a high level of general trust have more opportunities to participate in post-disaster rebuilding activities and therefore displayed increased positive psychological well-being. Whereas, building general trust in the process of post-disaster reconstruction may be difficult, especially for survivors who are female, low income, disabled, and have experienced a serious loss of house and property (see Table A2 in Appendix B). 

The interaction tests provided novel evidence to understand how media exposure affects PTSD symptoms differently between adult survivors who have high vs. low levels of general trust. We hypothesized that media exposure would act as a coping strategy to reduce uncertainty during the disaster. Previous studies indicated a key motivation of seeking information is the negative emotions that are sparked during a disaster [27,28]. Most media coverage focused on real-time rescue situations and casualties. High trust survivors viewed media coverage and obtained adequate mental compensations according to the selective exposure hypothesis, which emphasizes that people prefer to receive information that is consistent with their attitudes [64]. Correspondingly, survivors who had a low level of general trust were more likely to seek negative information about the disaster relief system and post-disaster rebuilding activities via rumors or gossips. Hence, future mental health promotion programs should incorporate strategies that can not only use media coverage to deliver supporting information, but also develop a social trust system that can help survivors access media effectively and obtain potential psychological benefits.

This study had some limitations. First, the cross-sectional design limits our ability to infer causation regarding long-term PTSD. Future studies may consider a panel design to collect longitudinal data. Second, although we followed suggestions from previous studies [30,49,50,51], two one-item measures to measure general trust and media exposure may not fully capture the multidimensional aspects of these constructs. Although it is difficult for participants to recall detailed media exposure from 10 years ago, diverse media platforms may play distinct roles in forming long-term mental disorders. Future studies may consider this point using a similar study design and develop a valid measurement based on some sophisticated measurement, such as the Media Attentiveness Scale [65]. Also, as we discussed, the low-trust and high-trust survivors might selectively expose themselves to different media contents or selectively internalize different information from the same media contents. Future study may concern the measurement of survivors’ media preferences and their perceived media credibility during the post-disaster period. Third, although we surveyed people from six counties to provide disaster area diversity, participants were not recruited via a random sampling method. This may weaken the overall representativeness of this study. Lastly, PTSD symptoms were measured using the PCL-C, which is not a clinical diagnostic method. As one previous study suggested, future studies may consider strengthening study validity and credibility via adopting a clinical diagnostic measurement [16].

## 5. Conclusions

In summary, we found a significant association between general trust and long-term PTSD symptoms among adult survivors 10 years after the 5.12 Wenchuan earthquake. General trust moderated linkages between exposure to media coverage about the disaster during the earthquake and long-term PTSD symptoms, indicating that media exposure can alleviate PTSD for high-trust survivors, but aggravate PTSD for low-trust survivors. These results suggest that general trust building should be considered in future post-disaster construction activities. 

Furthermore, building general trust for victims and first responders cannot be separated from governmental interventions. As Steinhardt found, in Chinese society, general trust was directly contributed by the institutional confidence, which refers to the confidence in the civil service and courts and the perceived degree of corruption in local governments [66]. These imply that when a local government responds slowly to a disaster, dissatisfaction and discontent toward it can increase and this could weaken general trust. Moreover, disaster-affected individuals may be more competitive over limited resources, resulting in conflicts among them. Consequently, the net effects of disasters on general trust levels may vary based on the speed of government responses to disasters and the active support for the victims from local communities. Both neighborhood level and institutional level interventions should be allocated appropriately.

## Figures and Tables

**Table 1:** Descriptive statistics.

Variable	Obs	Mean	Std.Dev.	Min	Max
***PTSD symptoms***					
PCL-C score	1000	2.364	0.626	1	4.412
Reexperiencing	1000	2.364	0.692	1	4.6
Avoidance	1000	2.374	0.673	1	4.571
***Arousal***	1000	2.349	0.726	1	4.4
***Independent variables***					
Media exposure	1000	3.765	0.985	1	5
General trust (0 = low trust)	1000	0.575	0.495	0	1
***Demographic and socioeconomic variables***					
Male	1000	0.435	0.496	0	1
Age	1000	45.618	11.628	28	74
Rural site	1000	0.557	0.497	0	1
Married	1000	0.827	0.378	0	1
Educational background (0 = primary school and below)					
Junior high school	1000	0.313	0.464	0	1
Senior high school	1000	0.277	0.448	0	1
Associate college and above	1000	0.253	0.435	0	1
Annual household income (0 = less than 40,000 RMB)					
40,000–59,999 RMB	1000	0.286	0.452	0	1
60,000–89,999 RMB	1000	0.255	0.436	0	1
90,000 RMB and above	1000	0.221	0.415	0	1
**Earthquake exposure**					
Being buried	1000	0.024	0.153	0	1
Being injured	1000	0.152	0.359	0	1
Being disabled	1000	0.018	0.133	0	1
Family died	1000	0.057	0.232	0	1
Family injured	1000	0.218	0.413	0	1
Family disabled	1000	0.061	0.239	0	1
Kinsfolk died	1000	0.138	0.345	0	1
Kinsfolk injured	1000	0.388	0.488	0	1
Kinsfolk disabled	1000	0.179	0.384	0	1
Acquaintance died	1000	0.197	0.398	0	1
Acquaintance injured	1000	0.423	0.494	0	1
Acquaintance disabled	1000	0.303	0.460	0	1
Witness to others’ bury	1000	0.130	0.336	0	1
Witness to others’ death	1000	0.232	0.422	0	1
Witness to others’ injury	1000	0.360	0.480	0	1
Loss of house and property (0 = mildly)					
Moderate	1000	0.420	0.494	0	1
Serious	1000	0.310	0.463	0	1

**Table 2:** Tobit model of predicting PTSD symptoms.

Variables	Model 1	Model 2	Model 3	Model 4	Model 5	Model 6	Model 7	Model 8
	PCL-C Score		Reexperiencing		Avoidance		Arousal	
***Independent variables***								
Media exposure		−0.01		0.01		−0.03		−0.00
		(0.02)		(0.02)		(0.02)		(0.02)
General trust (0 = low trust)		−0.12 **		−0.08 +		−0.13 **		−0.16 ***
		(0.04)		(0.04)		(0.04)		(0.05)
***Demographic and socioeconomic variables***								
Male	0.05	0.04	0.08 +	0.07	0.03	0.02	0.05	0.04
	(0.04)	(0.04)	(0.04)	(0.04)	(0.04)	(0.04)	(0.05)	(0.05)
Age	0.01 ***	0.01 ***	0.01 **	0.01 **	0.01 **	0.01 **	0.01 ***	0.01 ***
	(0.00)	(0.00)	(0.00)	(0.00)	(0.00)	(0.00)	(0.00)	(0.00)
Rural site	0.07	0.06	0.06	0.06	0.06	0.05	0.08	0.08
	(0.04)	(0.04)	(0.05)	(0.05)	(0.05)	(0.05)	(0.05)	(0.05)
Married	−0.07	−0.06	−0.14 *	−0.13 *	−0.03	−0.02	−0.05	−0.05
	(0.05)	(0.05)	(0.06)	(0.06)	(0.06)	(0.06)	(0.06)	(0.06)
Educational background (0 = primary school and below)								
Junior high school	0.13 *	0.12 +	0.12	0.11	0.12 +	0.12	0.18 *	0.16 *
	(0.07)	(0.07)	(0.07)	(0.07)	(0.07)	(0.07)	(0.08)	(0.08)
Senior high school	0.25 **	0.24 **	0.23 *	0.22 *	0.24 **	0.23 **	0.30 **	0.28 **
	(0.08)	(0.08)	(0.09)	(0.09)	(0.09)	(0.09)	(0.10)	(0.09)
Associate college and above	0.40 ***	0.40 ***	0.32 **	0.32 **	0.40 ***	0.40 ***	0.48 ***	0.48 ***
	(0.09)	(0.09)	(0.10)	(0.10)	(0.10)	(0.10)	(0.11)	(0.11)
Annual household income (0 = less than 40,000 RMB)								
40,000–59,999 RMB	−0.10 +	−0.09	−0.10	−0.09	−0.11 +	−0.09	−0.08	−0.07
	(0.06)	(0.06)	(0.06)	(0.06)	(0.06)	(0.06)	(0.07)	(0.07)
60,000–89,999 RMB	−0.22 ***	−0.20 ***	−0.22 **	−0.21 **	−0.22 **	−0.21 **	−0.22 **	−0.20 **
	(0.06)	(0.06)	(0.07)	(0.07)	(0.07)	(0.07)	(0.08)	(0.07)
90,000 RMB and above	−0.12 +	−0.10	−0.05	−0.04	−0.18 *	−0.15 *	−0.11	−0.09
	(0.07)	(0.07)	(0.08)	(0.08)	(0.08)	(0.08)	(0.08)	(0.08)
***Earthquake exposure***								
Being buried	0.07	0.05	−0.01	−0.02	0.06	0.04	0.17	0.15
	(0.12)	(0.12)	(0.14)	(0.14)	(0.14)	(0.14)	(0.15)	(0.15)
Being injured	−0.13 *	−0.13 *	−0.10	−0.11	−0.14 *	−0.14 *	−0.17 *	−0.17 *
	(0.06)	(0.06)	(0.06)	(0.06)	(0.06)	(0.06)	(0.07)	(0.07)
Being disabled	0.10	0.05	0.02	−0.01	0.19	0.14	0.03	−0.03
	(0.14)	(0.14)	(0.16)	(0.16)	(0.16)	(0.16)	(0.18)	(0.18)
Family died	0.05	0.05	0.03	0.03	0.06	0.07	0.02	0.03
	(0.09)	(0.09)	(0.10)	(0.10)	(0.10)	(0.10)	(0.11)	(0.11)
Family injured	0.12 *	0.11 *	0.19 **	0.19 **	0.06	0.06	0.12 +	0.12 +
	(0.05)	(0.05)	(0.06)	(0.06)	(0.06)	(0.06)	(0.07)	(0.06)
Family disabled	−0.12	−0.12	−0.17 +	−0.16 +	−0.08	−0.08	−0.14	−0.14
	(0.08)	(0.08)	(0.09)	(0.09)	(0.09)	(0.09)	(0.10)	(0.10)
Kinsfolk died	−0.14 *	−0.14 *	−0.11 +	−0.12 +	−0.12 +	−0.12 +	−0.19 **	−0.20 **
	(0.06)	(0.06)	(0.07)	(0.07)	(0.06)	(0.06)	(0.07)	(0.07)
Kinsfolk injured	−0.15 ***	−0.15 ***	−0.16 ***	−0.16 ***	−0.12 **	−0.13 **	−0.17 ***	−0.17 ***
	(0.04)	(0.04)	(0.05)	(0.05)	(0.05)	(0.05)	(0.05)	(0.05)
Kinsfolk disabled	−0.06	−0.07	−0.11 +	−0.11 +	−0.02	−0.04	−0.09	−0.10
	(0.05)	(0.05)	(0.06)	(0.06)	(0.06)	(0.06)	(0.07)	(0.07)
Acquaintance died	−0.12 *	−0.11 *	−0.06	−0.05	−0.14 **	−0.13 *	−0.14 *	−0.13 *
	(0.05)	(0.05)	(0.05)	(0.05)	(0.05)	(0.05)	(0.06)	(0.06)
Acquaintance injured	−0.01	−0.01	0.06	0.07	−0.05	−0.04	−0.04	−0.03
	(0.04)	(0.04)	(0.05)	(0.05)	(0.04)	(0.04)	(0.05)	(0.05)
Acquaintance disabled	0.04	0.04	0.04	0.04	0.05	0.05	0.05	0.05
	(0.04)	(0.04)	(0.05)	(0.05)	(0.05)	(0.05)	(0.05)	(0.05)
Witness to others’ burial	−0.11 +	−0.11 +	−0.10	−0.10	−0.13 *	−0.13 *	−0.11	−0.11
	(0.06)	(0.06)	(0.07)	(0.07)	(0.06)	(0.06)	(0.07)	(0.07)
Witness to others’ death	−0.09 +	−0.09 +	−0.06	−0.06	−0.10 *	−0.09 +	−0.12 *	−0.11 *
	(0.05)	(0.05)	(0.05)	(0.05)	(0.05)	(0.05)	(0.06)	(0.06)
Witness to others’ injury	−0.05	−0.05	−0.07	−0.07	−0.06	−0.06	−0.02	−0.02
	(0.04)	(0.04)	(0.05)	(0.05)	(0.05)	(0.05)	(0.05)	(0.05)
Loss of house and property (0 = mildly)								
Moderate	0.32 ***	0.32 ***	0.31 ***	0.31 ***	0.32 ***	0.32 ***	0.36 ***	0.35 ***
	(0.05)	(0.05)	(0.05)	(0.05)	(0.05)	(0.05)	(0.06)	(0.06)
Serious	0.42 ***	0.41 ***	0.40 ***	0.39 ***	0.41 ***	0.39 ***	0.49 ***	0.48 ***
	(0.05)	(0.05)	(0.06)	(0.06)	(0.06)	(0.06)	(0.07)	(0.07)
Constant	1.76 ***	1.86 ***	1.83 ***	1.83 ***	1.79 ***	1.96 ***	1.57 ***	1.67 ***
	(0.17)	(0.18)	(0.19)	(0.21)	(0.19)	(0.20)	(0.21)	(0.22)
Observations	1000	1000	1000	1000	1000	1000	1000	1000
df	27	29	27	29	27	29	27	29
chi2	162	172	135.8	139	132	144.3	143.3	154.5
Log likelihood	-882.9	−877.9	−1013	−1011	−984.3	−978.2	−1087	−1081
PR2	0.0841	0.0892	0.0628	0.0643	0.0629	0.0687	0.0619	0.0667

Standard errors in parentheses; *** *p* < 0.001, ** *p* < 0.01, * *p* < 0.05, + *p* < 0.1.

**Table 3:** Interaction effects of media exposure and general trust on PTSD symptoms.

Variables	Model 1	Model 2	Model 3	Model 4
	PCL-C Score	Reexperiencing	Avoidance	Arousal
***Interaction Item***				
Media exposure × General trust	−0.07 +	−0.06	−0.06	−0.10 *
	**(0.04)**	**(0.04)**	**(0.04)**	**(0.05)**
***Independent variables***				
Media exposure	0.03	0.05	0.00	0.05
	(0.03)	(0.03)	(0.03)	(0.04)
General trust (0 = low trust)	0.14	0.15	0.08	0.21
	(0.15)	(0.17)	(0.16)	(0.18)
***Demographic and socioeconomic variables***				
Male	0.04	0.06	0.02	0.03
	(0.04)	(0.04)	(0.04)	(0.05)
Age	0.01 ***	0.01 **	0.01 **	0.01 ***
	(0.00)	(0.00)	(0.00)	(0.00)
Rural site	0.06	0.06	0.05	0.08
	(0.04)	(0.05)	(0.05)	(0.05)
Married	−0.06	−0.14 *	−0.02	−0.05
	(0.05)	(0.06)	(0.06)	(0.06)
Educational background (0 = primary school and below)				
Junior high school	0.12 +	0.11	0.11	0.16 *
	(0.06)	(0.07)	(0.07)	(0.08)
Senior high school	0.24 **	0.22 *	0.23 **	0.29 **
	(0.08)	(0.09)	(0.09)	(0.09)
Associate college and above	0.40 ***	0.32 **	0.40 ***	0.48 ***
	(0.09)	(0.10)	(0.10)	(0.11)
Annual household income (0 = less than 40,000 RMB)				
40,000–59,999 RMB	−0.08	−0.09	−0.09	−0.06
	(0.06)	(0.06)	(0.06)	(0.07)
60,000–89,999 RMB	−0.20 **	−0.21 **	−0.20 **	−0.20 **
	(0.06)	(0.07)	(0.07)	(0.07)
90,000 RMB and above	−0.09	−0.03	−0.15 +	−0.08
	(0.07)	(0.08)	(0.08)	(0.08)
***Earthquake exposure***				
Being buried	0.06	−0.02	0.05	0.16
	(0.12)	(0.14)	(0.14)	(0.15)
Being injured	−0.14 *	−0.11 +	−0.14 *	−0.17 *
	(0.06)	(0.06)	(0.06)	(0.07)
Being disabled	0.04	−0.01	0.14	−0.03
	(0.14)	(0.16)	(0.16)	(0.17)
Family died	0.06	0.04	0.08	0.04
	(0.09)	(0.10)	(0.10)	(0.11)
Family injured	0.12 *	0.20 **	0.06	0.13 +
	(0.05)	(0.06)	(0.06)	(0.06)
Family disabled	−0.12	−0.16 +	−0.08	−0.14
	(0.08)	(0.09)	(0.09)	(0.10)
Kinsfolk died	−0.14 *	−0.12 +	−0.12 +	−0.20 **
	(0.06)	(0.07)	(0.06)	(0.07)
Kinsfolk injured	−0.16 ***	−0.16 ***	−0.13 **	−0.18 ***
	(0.04)	(0.05)	(0.05)	(0.05)
Kinsfolk disabled	−0.08	−0.12 +	−0.04	−0.11
	(0.05)	(0.06)	(0.06)	(0.07)
Acquaintance died	−0.11 *	−0.05	−0.12 *	−0.13 *
	(0.05)	(0.05)	(0.05)	(0.06)
Acquaintance injured	−0.01	0.06	−0.05	−0.03
	(0.04)	(0.05)	(0.04)	(0.05)
Acquaintance disabled	0.04	0.04	0.05	0.05
	(0.04)	(0.05)	(0.05)	(0.05)
Witness to others’ burial	−0.11 +	−0.10	−0.13 *	−0.11
	(0.06)	(0.07)	(0.06)	(0.07)
Witness to others’ death	−0.09 +	−0.06	−0.10 +	−0.12 *
	(0.05)	(0.05)	(0.05)	(0.06)
Witness to others’ injury	−0.05	−0.07	−0.05	−0.02
	(0.04)	(0.05)	(0.05)	(0.05)
Loss of house and property (0 = mildly)				
Moderate	0.32 ***	0.31 ***	0.32 ***	0.35 ***
	(0.05)	(0.05)	(0.05)	(0.06)
Serious	0.41 ***	0.40 ***	0.39 ***	0.48 ***
	(0.05)	(0.06)	(0.06)	(0.07)
Constant	1.71 ***	1.70 ***	1.84 ***	1.46 ***
	(0.20)	(0.23)	(0.22)	(0.24)
Observations	1000	1000	1000	1000
Df	30	30	30	30
chi2	175.3	141	146.2	159
Log likelihood	−876.2	−1010	−977.3	−1079
PR2	0.0909	0.0652	0.0696	0.0686

Standard errors in parentheses; *** *p* < 0.001, ** *p* < 0.01, * *p* < 0.05, + *p* < 0.1.

## Appendix A

**Table A1:** Tobit model of predicting PTSD symptoms for low trust and high trust participants, respectively.

Variables	Model 1	Model 2	Model 3	Model 4	Model 5	Model 6	Model 7	Model 8
	Low Trust				High Trust			
	PCL-C Score	Reexperiencing	Avoidance	Arousal	PCL-C Score	Reexperiencing	Avoidance	Arousal
***Independent variables***								
Media exposure	0.04	0.07 *	0.02	0.06 +	−0.05 +	−0.03	−0.06 *	−0.06
	(0.03)	(0.03)	(0.03)	(0.03)	(0.03)	(0.03)	(0.03)	(0.03)
***Demographic and socioeconomic variables***								
Male	−0.00	0.02	0.01	−0.04	0.08	0.13 *	0.03	0.11 +
	(0.06)	(0.06)	(0.06)	(0.06)	(0.05)	(0.06)	(0.06)	(0.06)
Age	0.01 *	0.01 +	0.01 +	0.01 **	0.01 *	0.01	0.01 *	0.01 +
	(0.00)	(0.00)	(0.00)	(0.00)	(0.00)	(0.00)	(0.00)	(0.00)
Rural site	0.10	0.10	0.06	0.15 +	0.05	0.05	0.05	0.03
	(0.06)	(0.07)	(0.07)	(0.07)	(0.06)	(0.07)	(0.06)	(0.07)
Married	−0.11	−0.17 *	−0.09	−0.09	−0.01	−0.09	0.05	−0.02
	(0.07)	(0.08)	(0.08)	(0.08)	(0.07)	(0.08)	(0.07)	(0.09)
Educational background (0 = primary school and below)								
Junior high school	0.14	0.06	0.12	0.24 *	0.09	0.13	0.07	0.10
	(0.10)	(0.11)	(0.11)	(0.11)	(0.09)	(0.10)	(0.10)	(0.11)
Senior high school	0.31 **	0.22 +	0.29 *	0.44 ***	0.13	0.16	0.13	0.11
	(0.11)	(0.13)	(0.13)	(0.13)	(0.10)	(0.12)	(0.11)	(0.13)
Associate college and above	0.57 ***	0.36 *	0.56 ***	0.80 ***	0.19	0.20	0.21	0.19
	(0.13)	(0.15)	(0.15)	(0.15)	(0.12)	(0.14)	(0.13)	(0.15)
Annual household income (0 = less than 40,000 RMB)								
40,000–59,999 RMB	−0.15 +	−0.14	−0.21 *	−0.08	−0.02	−0.02	0.02	−0.06
	(0.08)	(0.09)	(0.09)	(0.09)	(0.08)	(0.09)	(0.08)	(0.10)
60,000–89,999 RMB	−0.31 ***	−0.39 ***	−0.34 ***	−0.20 *	−0.07	−0.03	−0.04	−0.19 +
	(0.08)	(0.10)	(0.10)	(0.10)	(0.09)	(0.10)	(0.09)	(0.11)
90,000 RMB and above	−0.10	−0.08	−0.16	−0.00	0.01	0.11	−0.01	−0.07
	(0.10)	(0.12)	(0.11)	(0.12)	(0.09)	(0.11)	(0.10)	(0.12)
***Earthquake exposure***								
Being buried	0.08	−0.04	0.12	0.15	0.02	−0.03	−0.01	0.14
	(0.15)	(0.17)	(0.17)	(0.18)	(0.20)	(0.23)	(0.22)	(0.25)
Being injured	−0.16 *	−0.16 +	−0.17 *	−0.14	−0.10	−0.01	−0.09	−0.21 *
	(0.08)	(0.09)	(0.09)	(0.09)	(0.08)	(0.09)	(0.09)	(0.10)
Being disabled	0.03	−0.07	0.12	−0.02	0.58	0.56	0.77 +	0.31
	(0.15)	(0.17)	(0.17)	(0.17)	(0.42)	(0.48)	(0.46)	(0.52)
Family died	0.17	0.10	0.15	0.26 +	−0.05	−0.02	0.02	−0.23
	(0.12)	(0.13)	(0.13)	(0.14)	(0.13)	(0.15)	(0.14)	(0.16)
Family injured	0.19 *	0.30 ***	0.11	0.19 *	0.08	0.11	0.01	0.15
	(0.07)	(0.08)	(0.08)	(0.09)	(0.08)	(0.09)	(0.09)	(0.10)
Family disabled	−0.04	−0.09	−0.02	−0.01	−0.19 +	−0.23 +	−0.14	−0.25 +
	(0.12)	(0.14)	(0.14)	(0.14)	(0.11)	(0.13)	(0.12)	(0.14)
Kinsfolk died	−0.06	−0.02	−0.05	−0.13	−0.23 **	−0.20 *	−0.20 *	−0.32 **
	(0.08)	(0.09)	(0.09)	(0.09)	(0.08)	(0.09)	(0.09)	(0.10)
Kinsfolk injured	−0.17 **	−0.21 **	−0.14 *	−0.18 **	−0.14 *	−0.11 +	−0.13 *	−0.16 *
	(0.06)	(0.07)	(0.07)	(0.07)	(0.06)	(0.07)	(0.06)	(0.07)
Kinsfolk disabled	−0.02	0.00	−0.01	−0.05	−0.14 +	−0.22 **	−0.07	−0.16 +
	(0.08)	(0.09)	(0.09)	(0.09)	(0.07)	(0.08)	(0.08)	(0.09)
Acquaintance died	−0.24 ***	−0.22 **	−0.25 **	−0.28 **	−0.03	0.05	−0.06	−0.04
	(0.07)	(0.08)	(0.08)	(0.09)	(0.06)	(0.07)	(0.07)	(0.08)
Acquaintance injured	−0.03	0.03	−0.07	−0.05	0.01	0.09	−0.03	−0.02
	(0.06)	(0.07)	(0.07)	(0.07)	(0.05)	(0.06)	(0.06)	(0.07)
Acquaintance disabled	0.02	−0.02	0.04	0.01	0.03	0.03	0.01	0.05
	(0.06)	(0.07)	(0.07)	(0.07)	(0.06)	(0.06)	(0.06)	(0.07)
Witness to others’ burial	−0.03	−0.10	0.02	−0.02	−0.13 +	−0.08	−0.19 *	−0.14
	(0.09)	(0.11)	(0.10)	(0.11)	(0.07)	(0.08)	(0.08)	(0.09)
Witness to others’ death	−0.16 *	−0.14 +	−0.19 *	−0.16 +	−0.03	−0.00	−0.03	−0.07
	(0.07)	(0.08)	(0.08)	(0.08)	(0.06)	(0.07)	(0.07)	(0.08)
Witness to others’ injury	0.02	−0.03	0.03	0.04	−0.08	−0.09	−0.10	−0.02
	(0.06)	(0.07)	(0.07)	(0.07)	(0.06)	(0.07)	(0.07)	(0.07)
Loss of house and property (0 = mildly)								
Moderate	0.16 *	0.13	0.19 *	0.15 +	0.39 ***	0.40 ***	0.37 ***	0.46 ***
	(0.08)	(0.09)	(0.09)	(0.09)	(0.06)	(0.07)	(0.07)	(0.08)
Serious	0.22 **	0.22 *	0.21 *	0.21 *	0.56 ***	0.51 ***	0.54 ***	0.70 ***
	(0.08)	(0.09)	(0.09)	(0.09)	(0.07)	(0.08)	(0.08)	(0.09)
Constant	1.81 ***	1.89 ***	2.01 ***	1.43 ***	1.86 ***	1.79 ***	1.88 ***	1.84 ***
	(0.26)	(0.30)	(0.30)	(0.31)	(0.25)	(0.28)	(0.27)	(0.31)
Observations	425	425	425	425	575	575	575	575
df	28	28	28	28	28	28	28	28
chi2	97.87	89.84	78.43	87.40	108.7	91	91.13	104.4
Log likelihood	−347.4	−404.5	−397.5	−416.8	−504.3	−582	−558.5	−634
PR2	0.123	0.100	0.0898	0.0949	0.0973	0.0725	0.0754	0.0761

Standard errors in parentheses; *** *p* < 0.001, ** *p* < 0.01, * *p* < 0.05, + *p* < 0.1.

## Appendix B

**Table A2:** Probit model of predicting high trust by demographic and socioeconomic variables and disaster exposure variables.

Variables	High Trust (1 = yes)
***Demographic and socioeconomic variables***	
Male	−0.26 **
	(0.08)
Age	0.00
	(0.01)
Rural site	−0.06
	(0.10)
Married	0.09
	(0.11)
Educational background (0 = primary school and below)	
Junior high school	−0.24 +
	(0.14)
Senior high school	−0.23
	(0.17)
Associate college and above	0.01
	(0.20)
Annual household income (0 = less than 40,000 RMB)	
40,000–59,999 RMB	0.20 +
	(0.12)
60,000–89,999 RMB	0.23 +
	(0.14)
90,000 RMB and above	0.36 *
	(0.15)
***Earthquake exposure***	
Being buried	−0.37
	(0.28)
Being injured	−0.01
	(0.12)
Being disabled	−1.33 **
	(0.41)
Family died	0.12
	(0.19)
Family injured	−0.04
	(0.12)
Family disabled	0.10
	(0.18)
Kinsfolk died	−0.06
	(0.13)
Kinsfolk injured	−0.06
	(0.09)
Kinsfolk disabled	−0.17
	(0.12)
Acquaintance died	0.15
	(0.11)
Acquaintance injured	0.09
	(0.09)
Acquaintance disabled	−0.06
	(0.09)
Witness to others’ burial	0.09
	(0.13)
Witness to others’ death	0.03
	(0.10)
Witness to others’ injury	−0.02
	(0.10)
Loss of house and property (0 = mildly)	
Moderate	−0.14
	(0.11)
Serious	−0.29 *
	(0.12)
Constant	0.19
	(0.37)
Observations	1000
df	27
chi2	72.94
Log likelihood	−645.4
PR2	0.0535

Standard errors in parentheses; *** *p* < 0.001, ** *p* < 0.01, * *p* < 0.05, + *p* < 0.1.
